# Quantitative Magnetic Resonance Imaging to Assess Progression and Rupture Risk of Aortic Aneurysms: A Scoping Review

**DOI:** 10.1177/15266028231204830

**Published:** 2023-10-18

**Authors:** Eva Aalbregt, Lotte Rijken, Aart Nederveen, Pim van Ooij, Kak Khee Yeung, Vincent Jongkind

**Affiliations:** 1 Department of Surgery, Amsterdam UMC location Vrije Universiteit Amsterdam, Amsterdam, The Netherlands; 2 Department of Radiology and Nuclear Medicine, Amsterdam UMC location University of Amsterdam, Amsterdam, The Netherlands; 3 Amsterdam Cardiovascular Sciences, Amsterdam, The Netherlands; 4 Amsterdam UMC, location AMC, Amsterdam, The Netherlands

**Keywords:** abdominal aortic aneurysm, thoracic aortic aneurysm, quantitative magnetic resonance imaging, molecular imaging, aortic rupture, biomarkers, flow, 3D imaging

## Abstract

**Purpose:**

In current practice, the diameter of an aortic aneurysm is utilized to estimate the rupture risk and decide upon timing of elective repair, although it is known to be imprecise and not patient-specific. Quantitative magnetic resonance imaging (MRI) enables the visualization of several biomarkers that provide information about processes within the aneurysm and may therefore facilitate patient-specific risk stratification. We performed a scoping review of the literature on quantitative MRI techniques to assess aortic aneurysm progression and rupture risk, summarized these findings, and identified knowledge gaps.

**Methods:**

Literature concerning primary research was of interest and the medical databases PubMed, Scopus, Embase, and Cochrane were systematically searched. This study used the PRISMA protocol extension for scoping reviews. Articles published between January 2010 and February 2023 involving animals and/or humans were included. Data were extracted by 2 authors using a predefined charting method.

**Results:**

A total of 1641 articles were identified, of which 21 were included in the scoping review. Quantitative MRI-derived biomarkers were categorized into hemodynamic (8 studies), wall (5 studies) and molecular biomarkers (8 studies). Fifteen studies included patients and/or healthy human subjects. Animal models were investigated in the other 6 studies. A cross-sectional study design was the most common, whereas 5 animal studies had a longitudinal component and 2 studies including patients had a prospective design. A promising hemodynamic biomarker is wall shear stress (WSS), which is estimated based on 4D-flow MRI. Molecular biomarkers enable the assessment of inflammatory and wall deterioration processes. The ADAMTS4-specific molecular magnetic resonance (MR) probe showed potential to predict abdominal aortic aneurysm (AAA) formation and rupture in a murine model. Wall biomarkers assessed using dynamic contrast-enhanced (DCE) MRI showed great potential for assessing AAA progression independent of the maximum diameter.

**Conclusion:**

This scoping review provides an overview of quantitative MRI techniques studied and the biomarkers derived from them to assess aortic aneurysm progression and rupture risk. Longitudinal studies are needed to validate the causal relationships between the identified biomarkers and aneurysm growth, rupture, or repair. In the future, quantitative MRI could play an important role in the personalized risk assessment of aortic aneurysm rupture.

**Clinical Impact:**

The currently used maximum aneurysm diameter fails to accurately assess the multifactorial pathology of an aortic aneurysm and precisely predicts rupture in a patient-specific manner. Quantitative magnetic resonance imaging (MRI) enables the detection of various quantitative parameters involved in aneurysm progression and subsequent rupture. This scoping review provides an overview of the studied quantitative MRI techniques, the biomarkers derived from them, and recommendations for future research needed for the implementation of these biomarkers. Ultimately, quantitative MRI could facilitate personalized risk assessment for patients with aortic aneurysms, thereby reducing untimely repairs and improving rupture prevention.

## Introduction

Aortic aneurysms can be divided into abdominal aortic aneurysms (AAA) and thoracic aortic aneurysms (TAAs) based on their location. Aortic aneurysms are often asymptomatic until rupture, which is a devastating event with a mortality rate up to 80%.^1–3^ Elective surgery can be performed to prevent ruptures. In current practice, the diameter of an aortic aneurysm is used to estimate the risk of rupture and decide upon timing of elective repair. However, aneurysm diameter is known to be imprecise in predicting patient-specific risk of rupture.^4^ Consequently, patients with an aortic aneurysm below the operative diameter may still rupture,^2^ particularly women,^5^ whereas some aneurysms of extreme size do not rupture.^6^ The aortic size index enables a more patient-specific risk assessment by adjusting for body mass but is not a better predictor of AAA development and aortic growth compared with diameter alone.^7^ Moreover, aneurysm diameter and aortic size index are static measures that provide no information regarding the regional forces acting on the aortic wall. With quantitative imaging, both the hemodynamic forces responsible for stress on the aortic wall and processes that weaken that wall can be assessed.^8,9^ Consequently, there is growing interest in quantitative imaging parameters as novel predictors of aortic aneurysm progression and risk of rupture.

Quantitative imaging enables the measurement of physical or chemical variables quantified in numeric physical units. Quantitative imaging parameters can be measured with positron emission tomography (PET), computed tomography (CT), ultrasound, or MRI. PET enables the visualization of the inflammatory progress of aortic aneurysms but requires the injection of a radioactive compound and has limited spatial resolution^10,11^ CT has good spatial resolution but makes use of ionizing radiation and requires nephrotoxic contrast agents.^12^ Ultrasound imaging is relatively cheap and widely available but operator dependent and has lower resolution with increased depth.^13^

Quantitative MRI enables the measurement of quantitative parameters in addition to conventional qualitative anatomical information^14^ does not require ionizing radiation. Examples of quantitative MRI parameters are extent of turbulent flow within the aneurysm and perfusion of the dilated wall.^15^ MRI uses strong magnetic fields and radiofrequency pulses to generate images. It is a versatile imaging technique that has developed tremendously over the last few years.^2^ This led to the introduction of several potential biomarkers for aneurysm progression and rupture based on various disease mechanisms.^16–18^

Magnetic resonance biomarkers can be categorized as hemodynamic, molecular, and wall biomarkers. Hemodynamic biomarkers include those that quantify the dynamics of flowing blood and parameters that arise from that such as shear stress. Molecular biomarkers target specific molecular processes within and surrounding an aneurysm, such as inflammation. Finally, wall biomarkers visualize properties of the wall other than molecular properties such as stiffness and perfusion.

Magnetic resonance imaging studies have been conducted in humans and animal models. Several animal aneurysm models have been developed including genetic, surgical, and chemical models. In animal models we can follow aneurysms over time to assess their progression until rupture.^19,20^ In addition, motion artifacts and long scan durations are less of an issue, because animals are often anesthetized during scanning.^21^ Although animal models cannot perfectly mimic human pathophysiology, they provide knowledge the needed to better understand molecular and cellular mechanisms underlying disease.^22^

Currently, it is unclear which MRI-derived biomarkers are particularly promising for aortic aneurysm rupture risk prediction and subsequent clinical implementation. Therefore, a systematic review of the literature, designed as a scoping review, is required to describe and categorize the existing body of literature. The aim of this scoping review was to determine the extent of research performed both in humans and animals on quantitative MRI of aortic aneurysms, particularly focusing on progression and rupture prediction, summarizing these findings, identifying knowledge gaps, and describing the outlook for future research.

## Materials and Methods

### Search Strategy

This study is based on a scoping review of the existing literature performed according to the PRISMA protocol extension for scoping reviews.^23^ A scoping review can be described as the process of mapping relevant literature in a field of interest.^24,25^ The research question was defined prior to the systematic search and primary literature was of interest. Medical databases PubMed, Scopus, Embase, and Cochrane were searched to find relevant articles published between January 2010 and the date the search was conducted (February 22, 2023). Due to recent advancements in MRI research, studies conducted earlier than 2010 were considered outdated. The search strategy involved 3 blocks using a combination of medical subject headings (MeSH) and free text words regarding (1) MRI, (2) quantitative parameters, and (3) aortic aneurysms. Magnetic resonance imaging, quantitative MRI, and AAA are examples of used search terms. All aortic aneurysms were included within the search reasoning that biomarkers established in the thoracic aorta may be translated to the abdominal aorta and vice versa. In addition, cross-sectional studies involving animal models or investigating biomarker differences between healthy volunteers and patients with dilated aortas were included. Non-English publications, literature reviews, conference abstracts, and case reports were excluded. For detailed information about the search strategy we refer to the OSF Registries where the protocol was submitted doi.org/10.17605/OSF.IO/KDMYT. The full electronic search strategy as conducted in PubMed is given in the appendix. The search strategy was tailored to the specific requirements of the different databases.

### Study Selection

To remove duplicates, all identified articles were exported to EndNote. Subsequently, the resulting articles were uploaded to Rayyan (Rayyan Systems Inc., Cambridge, MA)^26^ for the screening of titles and abstracts. Two reviewers (E.A. and L.R.) independently selected studies for full-text review based on the title and abstract, and were blinded to each other’s decisions. Articles concerning cerebral or cardiac diseases, interventional or postoperative MRI, techniques not translatable to the clinic, and studies with outcomes without a link to aneurysm diameter, progression, rupture, or repair were excluded. Additional post hoc exclusion criteria were used, to assess the articles eligible for full-text review. Any disagreement between the 2 reviewers was resolved through a discussion with a third senior reviewer (V.J.). Backward citation chaining and hand searching were undertaken to find additional relevant articles by reviewing the reference lists of the included articles.

### Data Assessment

All articles were summarized by E.A. before data charting was executed in duplicates by E.A. and L.R. A data-charting form was jointly developed by the 2 reviewers to determine which variables to extract. The 2 reviewers independently charted the data, discussed the results and continuously updated the data-charting form in an iterative process. The following data were extracted: author information (title, author and year of publication); study population (animals, patients or healthy controls, number included); utilized MRI techniques and sequences; field strength of the utilized MRI scanner; quantitative biomarker studied and its target mechanism; study design, endpoints and conclusion. No extra or confirming data were obtained from investigators of the included studies. The extracted data were presented in a descriptive manner, summarized in tables, and not quantitatively assessed.

## Results

**Figure 1.**
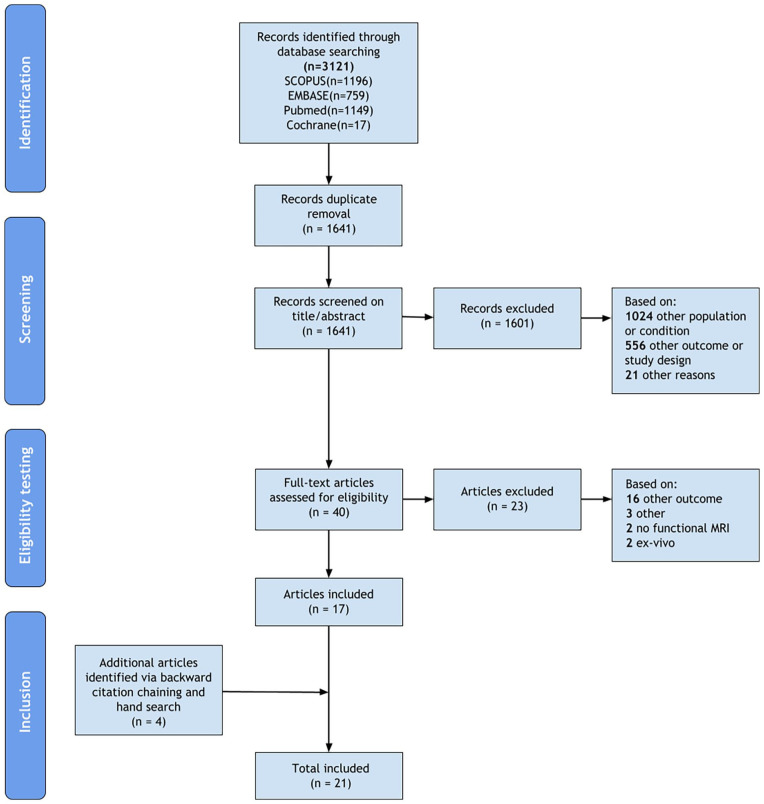
Flow diagram illustrating the conducted search strategy. After title/abstract screening, 21 records were excluded for other reasons: imaging technique other than magnetic resonance imaging, nonquantitative biomarker, outdated, and non-English publications. Three records were excluded after full-text review for other reasons: healthy volunteer study and interventional magnetic resonance study.

Figure 1 presents the flow diagram of the literature search. A total of 1641 articles were identified by the search, of which 40 remained for full text review after title/abstract screening. Twenty-three articles were excluded based on full text and 4 additional articles were identified through backward citation chaining and hand searching, resulting in 21 articles included in this scoping review.

Several quantitative MRI-based biomarkers have been described in the included articles. Eight articles involved a hemodynamic biomarker^8,18,27–32^ (Table 1), 5 articles involved a wall biomarker^9,33^–^36^ (Table 2), and 8 articles involved a molecular biomarker^16,21,37–42^ (Table 3). Only 1 study was conducted on an MR scanner with a nonclinical field strength of 9.4T.^21^ Other studies have been conducted using 1.5T or 3T scanners which are common in clinical practice. In addition, several different MRI techniques and sequences have been used to assess quantitative biomarkers. The study subjects included animal models, healthy volunteers and patients. Six studies (29%) included animal models,^9,16,21,37,38,42^ of which 2 studies also utilized excised human tissue for in vitro analyses.^38,42^ One of the animal studies investigated a wall biomarker in pigs^9^ whereas the other 5 focused on molecular biomarkers in a murine model.^16,21,37,38,42^ Wall stiffness is the only biomarker included in this scoping review that has been assessed in both animals and humans.^9,33^ Fifteen studies (71%) included patients. Of those, 2 studies included patients with Marfan syndrome (MFS),^28,32^ 3 studies included TAA patients,^18,27,32^ and 10 studies included AAA patients.^8,29,31,33–36^,^39–41^ Two studies^8,32^ used the term dilated aorta because not all included patients sufficed the definition of aneurysm. Fourteen of the included studies had a cross-sectional design without the endpoints of aneurysm progression, rupture or repair. Five animal studies had a (partly) longitudinal study design with endpoint growth and rupture caused death, with a follow-up of 4 weeks.^9,16,21,38,42^ In addition, 2 included human studies had a prospective study design^32,40^ with respective follow-up of over 2 years and 1 year. The assessed biomarkers were categorized and described below.

**Table 1.** Overview of Included Articles Concerning Hemodynamic Biomarkers.

Author	Publication year	MRI technique(s)	Field strength	Population	Biomarker	Longitudinal (component in) design	Study design and/or endpoint	Conclusion
Bürk et al	2012	4D-flow CMR based on RF-spoiled GRE, T_1_w 3D GRE	3T	Patients with AAo dilatation, N=33; age-matched volunteers, N=15; healthy young volunteers, N=15	3D blood flow patterns, WSS, OSI	No	Cross-sectional study whereby flow based biomarkers were compared between patients with aortic dilatation and controls	Increased AAo diameter was significantly correlated with more pronounced supra-physiologic-helix and vortex flow, a decrease in systolic WSS and an increase in OSI
Condemi et al	2019	4D-flow CMR based on RF-spoiled GRE	3T	AAo aneurysm patients planned for open surgical repair, N=10	WSS, TAWSS, flow-eccentricity, -helicity, -intensity and in vitro bulge inflation test	No	Cross-sectional study to combine CT, 4D-flow MRI and CFD models to analyze flow patterns in vivo, in vitro and in silico	In AAo aneurysms, relatively low TAWSS correlated with reduced aneurysm strength (measured in vitro by assessing rupture stretch)
Guala et al	2019	bSSFP cine, 4D-flow CMR based on PC	1.5T	Marfan patients, N=75; and healthy controls N=48	In-plane rotational flow, systolic flow reversal ratio, axial and circumferential WSS	No	Cross-sectional study to find early markers for thoracic aorta dilation in Marfan patients	Decreased rotational flow and circumferential WSS were present in thoracic aortas of Marfan patients with and without dilatation; axial and circumferential WSS were independently related to proximal descending aorta diameter in these patients
Korpela et al	2021	4D-flow CMR based on PC	1.5T	Patients with AAo dilatation, N=30	Flow displacement and WSS	Yes	Prospective clinical study with 1-year follow-up whereby aortic dilatation was correlated with changes in flow displacement and WSS	Flow displacement and decreased WSS seemed to be associated with growth of dilated AAo at 1-year follow-up (based on growth in 6 patients)
Takehara et al	2020	3D MRA, 2D PC cine, 4D-flow CMR	1.5T	Patients with abdominal aortic dilatation, N=18	Vortex flow, helical flow WSS and OSI	No	Cross-sectional study to measure nonlaminar flow and its derivatives in dilated abdominal aortas	Lower WSS and higher OSI are present in infrarenal aortic dilatations compared with upstream abdominal aorta due to more frequent and overt vortex and helical flow in dilated aortas
Trenti et al	2022	bSSFP, CE-CMRA, 4D-flow CMR	3T	AAA patients, N=18; age-matched controls, N=22; young subjects, N=23	WSS, OSI, RRT	No	Cross-sectional study to measure WSS, OSI and RRT in healthy volunteers compared with AAA patients	AAA patients have notably abnormal hemodynamic stresses in the entire aorta; RRT emerged as a powerful marker for altered hemodynamics in AAA
Van der Palen et al	2017	2D cine SSFP, CE-MRA, 4D-flow CMR	1.5T	Marfan syndrome patients N=25, healthy pediatric subjects N=21	3D blood flow velocity, mean systolic WSS, vortex flow, helix flow	No	Cross-sectional study to compare flow parameters in Marfan syndrome patients and healthy age-matched controls	Marfan patients presented altered aortic flow patterns and differences in WSS compared with healthy controls; an inverse relation between mean WSS and regional aortic diameter was found
Ziegler et al	2018	4D-flow CMR, CE-MRA, bSSFP	3T	Male AAA patients N=13	Flow stasis	No	Comparative study for different approaches of visualizing and quantifying aneurysmal flow stasis	Mean velocity analysis and particle travel distance analysis were more robust to velocity field distortions compared with computation of residence time; 4D-flow MRI can quantitatively assess flow stasis

Abbreviations: 2D, 2 dimensional; 3D, 3 dimensional; 4D, 4 dimensional; AAA, abdominal aortic aneurysm; AAo, ascending aorta; bSSFP, (balanced) steady-state free-precession; CE, contrast enhanced; CFD, computational fluid dynamics; CMR, cardiovascular magnetic resonance; CMRA, Cardiovascular magnetic resonance angiography; CT, computed tomography; GRE, gradient echo; MRA, magnetic resonance angiography; MRI, magnetic resonance imaging; OSI, oscillatory shear index; PC, phase contrast; RF, radiofrequency; RRT, relative residence time; (TA)WSS, (time-averaged) wall shear stress.

**Table 2.** Overview of Included Articles Concerning Wall Biomarkers.

Author	Publication year	MRI technique(s)	Field strength	Population	Biomarker	Longitudinal (component in) design	Study design and/or endpoint	Conclusion
Dong et al	2020	Cine GRE MRE	1.5T	Pigs with induced AAA divided into 3 cohorts 2-week (2W), 4-week (4W) and 4-week burst (4WB), N=31	Wall stiffness	No	Proof of concept study to find correlation between in vivo MRE derived AAA stiffness and 1. histopathology (all cohorts), 2. uniaxial tensile test (2W, 4W) and 3. burst testing (4WB)	MRE-derived AAA stiffness was higher compared with the distal aorta, correlated with histopathological analyses, and inversely correlated with wall strength expressed in peak stress and burst pressure
Kolipaka et al	2016	GRE MRE	3T	AAA patients with diameter between 3–10 cm, N=24; and healthy controls, N=12	Wall stiffness	No	Cross-sectional study to assess the correlations between aortic wall stiffness in AAA and several related parameters (diameter and ILT volume)	Wall stiffness was higher in AAA compared with the distal normal aorta and compared with healthy volunteers; AAA wall stiffness was independent of diameter and ILT volume
Kuzniar et al	2019	T_2_w FS Propeller, DWI, Dixon	3T	Patients with asymptomatic infrarenal AAA diameter > 4.5 cm, N=15	Combination of FDG uptake (PET (marker for inflammation) and MRI markers for inflammation such as LGE (marker for vascular leakage)	No	Cross-sectional study with retrospective growth assessment to investigate inflammation with FDG-PET and contrast enhanced MRI	Median diameter expansion rate was 3 mm in 12 months; the number of FDG hotspots and the presence of LGE in AAAs correlated with this aneurysm growth
Nguyen et al	2014	DCE-MRI with T_1_w FFE, T_1_w 3D TFE and bright blood	1.5T	AAA patients diameter > 3 cm, N=28	K^trans^; marker for increased microvasculature in AAA wall and AUC at 1 and 5 minutes	No	Proof of concept study, primary outcomes: feasibility and reproducibility of DCE-MRI in AAA	DCE-MRI of the AAA wall was reproducible; K^trans^ was moderately correlated with maximum diameter; no significant correlations were found between AUC and maximum diameter
Zhou et al	2023	DCE-MRI with 3D fast GRE Star-VIBE and bright blood	3T	Male AAA patients, N=27	K^trans^, AUC at 1 and 4 minutes and the slope of the initial contrast signal increase over time	No	Cross-sectional study with retrospective growth assessment whereby DCE-MRI-derived biomarkers are correlated with prior aneurysm growth	None of the DCE-MRI-derived biomarkers were associated with maximum diameter; contrast enhancement slope and AUC at 4 minutes were associated with recent AAA growth rate independent of maximum diameter

The article above the double line is an animal study.

Abbreviations: 3D, 3 dimensional; AAA, abdominal aortic aneurysm; AUC, area under the curve; CFD, computational fluid dynamics; CT, computed tomography; DCE, dynamic contrast enhanced; DWI, diffusion weighted imaging; ILT, intraluminal thrombus; FDG, fluorodeoxyglucose; FFE, fast field echo; FS, fat saturated; GRE, gradient echo; K^trans^, volume transfer constant; LGE, late gadolinium enhancement; MRE, magnetic resonance elastography; MRI, magnetic resonance imaging; PET, positron emission tomography; Star-VIBE, stack-of-stars volumetric interpolated breath-hold examination; T_1_w, T_1_-weighted; T_2_w, T_2_-weighted; TFE, turbo field echo.

**Table 3.** Overview of Included Articles Concerning Molecular Biomarkers.

Author	Publication year	MRI technique(s)	Field strength	Population	Biomarker	Longitudinal (component in) design	Study design and/or endpoint	Conclusion
Adams et al	2020	2D-TOF angiography, T_2_*w, IR with 2D Look-Locker sequence	3T	AngII-induced dissecting AAA ApoE−/− knockout mice, longitudinal set-up N=13 and week-by-week set-up, N=36	Dual probe technique: collagen-specific probe as a marker of collagen matrix degradation and iron oxide particles to target macrophage infiltration/inflammatory activity	Yes	Longitudinal set-up with rupture caused death as endpoint correlated with biomarkers, week-by-week set-up with endpoint aneurysm growth over 4 weeks	The dual probe technique, targeting both collagen-related ECM remodeling and inflammatory activity, was the most accurate predictor of AAA rupture
Bazeli et al	2010	PC MRA, T_1_w SE	1.5T	Lewis rats with elastase-induced AAAs, N=18	Gadolinium based contrast agent to target MMPs (P947)	No	Cross-sectional study to detect MMP activity based on MRI signal enhancement in induced AAAs	MRI with contrast agent P947 enabled the detection of MMP activity within the inflammatory wall of induced AAAs; signal enhancement was higher with P947 compared with reference contrast agents
Kaufmann et al	2022	3D GRE scout, 2D-TOF angiography, 2D TI scout, 3D IR FLASH	3T	ApoE−/− knockout mice, with AngII-induced AAA N=32, sham-operated mice N=10, excised ruptured human aortic aneurysm tissue N=3	MR probe to visualize the extracellular matrix enzyme ADAMTS4	Yes	Identifying ADAMTS4-specific MR probe and validating it’s potential in a cross-sectional, longitudinal and early detection study with an AAA mice model and endpoints growth and rupture caused death, ex vivo visualization of ADAMTS4 in human tissue	The ADAMTS4-specific MR probe could predict further AAA expansion in a murine model. Also, they could separate the mice by ADAMTS4- levels into 3 different groups: development of an AAA likely to rupture, AAA development and no AAA development
Klink et al	2011	Black-blood T_1_w SE, T_2_w SE, PDW SE	9.4T	C57BL/6 male mouse model with AngII induced AAA, N=25	Paramagnetic/fluorescent micellar nanoparticles functionalized with a collagen-binding protein (CNA-35)	Yes	Proof-of-concept experiment to detect and characterize (stable/unstable) AAAs in vivo based on CNA-35. Study with cross-sectional and longitudinal component with death as endpoint	Multisequence MRI enabled longitudinal monitoring of AAA progression; CNA-35 micelles have potential to discriminate stable from unstable AAA lesions that are likely to rapidly progress
Lavin et al	2019	Contrast enhanced angiography, LGE MRI, T1 mapping scans, 2D flow	3T	AngII-infused ApoE−/− mice with induced AAA, cross-sectional study N=55, treatment group N=10, longitudinal study N=19, excised human aortic aneurysm tissue N=4	(Tropo)elastin-specific contrast agent complexed with gadolinium (Gd-(T)ESMA)	Yes	Study to assess Gd-TESMA uptake in dilated abdominal aorta both with a cross-sectional set-up and longitudinal set-up with aortic expansion as endpoint, in vivo imaging with ex vivo validation	Gd-TESMA enhancement was confined to dilated aortic segments, and higher in and expressed more in dilating compared with nondilating aortas; tropoelastin accumulation in excised human tissue was identified with Gd-TESMA
Nchimi et al	2010	T_2_w TSE, T_2_*w and T_1_w GRE	1.5T	AAA patients with indication for surgery, N=15	SPIO particle contrast agent to target leukocyte phagocytic activity within thrombi	No	Cross-sectional study with SPIO-enhanced preoperative in vivo MR imaging, postoperative ex vivo MR imaging and laboratory analysis with zymography	SPIO uptake was mainly localized at the lumen-thrombus interface in high-risk AAAs, and correlated with the locations of leukocyte accumulation
Newby et al	2017	T_2_w TSE, multi-echo T_2_*w GRE	3T	AAA patients diameter > 4 cm, N=342	USPIO particle contrast agent to target tissue-resident macrophages (inflammation)	Yes	Prospective proof of concept phase 2 study with primary endpoint: aneurysm rupture or repair	Aneurysm repair or rupture was more frequent in patients with USPIO enhancement; USPIO enhancement could not independently predict aneurysm repair or rupture
Richards et al	2011	T_1_w and T_2_w TSE, multi-echo T_2_*w GRE	3T	Asymptomatic AAA patients diameter > 4 cm, N=29	USPIO particle contrast agent to target tissue-resident macrophages (inflammation)	No	Pilot study to correlate USPIO uptake to (1) cellular inflammation and (2) AAA growth rate determined based on 2 ultrasound scans; 1 before and 1 after MRI	AAA growth rate was threefold higher in patients with distinct areas of increased USPIO uptake compared with patients with no or nonspecific uptake

Articles above the double line involve animal models.

Abbreviations: 2D, 2 dimensional; 3D, 3 dimensional; AAA, abdominal aortic aneurysm; ADAMTS4, a disintegrin and metalloproteinase with thrombospondin motifs 4; AngII, angiotensin II; FLASH, fast low angle shot; GRE, gradient echo; IR, inversion recovery; LGE, late gadolinium enhancement; MMPs, matrix metalloproteinases; MRA, magnetic resonance angiography; MRI, magnetic resonance imaging; PC, phase contrast; PDW, proton density weighted; SE, spin echo; SPIO, superparamagnetic iron oxide; T_1_w, T_1_-weighted; T_2_w, T_2_-weighted; T_2_*w, T_2_*-weighted; TI, inversion time; TOF, time-of-flight; TSE, turbo spin echo; USPIO, ultrasmall superparamagnetic iron oxide.

### Hemodynamic Biomarkers

4D-flow MRI (time-resolved 3D phase-contrast MRI with 3-directional velocity encoding) can be used to measure flow velocity in 3 directions over time and assess hemodynamics.^8,18,27–30,32^ All hemodynamic biomarkers described below were assessed using 4D-flow MRI in human subjects. Hemodynamic biomarkers were studied in patients with TAA, AAA, and MFS. An overview of all included articles concerning hemodynamic parameters is given in Table 1.

#### Nonlaminar flow

Helical flow is the rotational motion of flow around the longitudinal axis of, or parallel to, the centerline of the vessel.^27^ Vortex flow is defined as particles circling around a point within the aorta deviating 90° or more from the centerline.^27,43^ Several studies have assessed helix and vortex flow in patients with aortic dilation using semi-quantitative grading scales and 4D-flow MRI data.^8,27,30^ The incidence and strength of these flow patterns were significantly higher in patients with dilated abdominal and thoracic aortas, than in age-matched controls, healthy volunteers and distal nondilated aortas and correlated with aneurysm diameter.^27,43^ Altered aortic flow patterns were observed in patients with MFS in the proximal descending aorta and ascending aorta compared with healthy volunteers.^30^ Another study reported decreased in-plane rotational flow in patients with MFS compared with healthy controls, even in MFS patients without thoracic aorta dilatation.^28^ The in-plane rotational flow quantifies the circumferential component of helical flow by isolating the revolving part of the velocity field within the plane.^28^ Comparing studies that assess nonlaminar flow is hindered by the lack of a standardized grading scale.

#### Wall shear stress

Wall shear stress (WSS) is the frictional tangential force per unit surface area exerted by the flowing blood on the vessel wall and vice versa. Endothelial cells in the aortic wall function as mechanoreceptors of flow velocity by detecting WSS.^44,45^ The responses of endothelial cells have been studied via bioengineering approaches in which cultured endothelial cells are subjected to shear stress in flow-loading devices.^45^ In response to abnormally low WSS, dysregulation of the anti-inflammatory and antioxidant mediators secreted by endothelial cells can lead to wall remodeling and dilatation.^27,46^

All included studies estimated WSS by assessing the near-wall-blood-flow velocity gradient.^8,18,27–30^ WSS was assessed in patients with TAA, AAA, and MFS discussed below in that order. Condemi et al^18^ included 10 patients with ascending aortic aneurysms who were scheduled for open surgical repair. During surgical repair, the aneurysm tissue was resected, thereby enabling a combination of in vitro and in vivo analyses. They defined the rupture stretch as the stretch needed to rupture the in vitro wall sample, which was calculated based on a bulge inflation test considering biaxial rupture.^18^ Rupture stretch is correlated with time-averaged (TA)WSS, which indicates that a relatively low TAWSS may impair aneurysm strength.^18^ Other studies reported significantly lower values of peak systolic WSS in AAA patients^29^ and patients with a dilated ascending aorta^27^ compared with age-matched controls and healthy volunteers. Moreover, WSS was lower in dilated aortic wall segments compared with nondilated regions in the same patients.^8^ The ascending aortic diameter seemed to inversely correlate with the peak systolic WSS.^27^ In a longitudinal study with 1-year follow-up and 30 included ascending aorta aneurysm patients, decreased WSS was associated with growth (median 2.1 mm [1.5–2.2 mm] in 6 patients).^32^

The 4D-flow MRI-derived WSS was also utilized to study aortic dilatation in patients with MFS. Reduced circumferential WSS was found in patients with MFS regardless of aortic dilation, which advocates for different hemodynamic effects in MFS. Moreover, WSS inversely correlated with regional diameter of the aneurysm.^28,31^

#### Oscillatory shear index

The oscillatory shear index (OSI) is a measure of the directional fluctuations of the WSS over the cardiac cycle, which is indicative of vascular remodeling and wall dysfunction.^46,47^ A high OSI may induce an inflammatory response including an enhancement of the vascular production of reactive oxygen species.^48^ Bürk et al^27^ found a correlation between the OSI and diameter in ascending aorta dilatations. Another study showed that the OSI in infrarenal AAAs was significantly higher than that in proximal nondilated aortic segments in the same patient.^8^ However, in a study comparing OSI in the infrarenal abdominal aorta of AAA patients, age-matched controls and young controls no differences were found between the groups.^29^

#### Relative residence time

The OSI can identify regions of flow reversal, but is insensitive to the magnitude of the WSS.^49^ Therefore, a combination of OSI and other WSS measures, such as relative residence time (RRT), may perform better.^29^ RRT is a combination of OSI and TAWSS.^49^ Regions of increased RRT values were associated with extensive elastin degradation^47^ and atherosclerotic lesions in a murine model.^50^ In a study including AAA patients, age-matched controls and healthy controls, RRT was identified as a marker for abnormal hemodynamics in AAAs and enabled the distinction between AAA patients and elderly controls; which was not possible based on OSI alone.^29^

#### Flow stasis

Flow stasis creates an environment favorable to platelet adhesion and thrombus formation which may lead to aneurysm growth and regional hypoxia.^51–53^ A single study assessed flow stasis quantification methods in male AAA patients without assessing the possible correlation between flow stasis and AAA progression.^31^

#### Flow displacement

Flow displacement (FD) is the distance between the center of the lumen and the center of forward blood flow velocity within the lumen. Thirty patients with an ascending aorta dilatation were scanned twice with a 1-year interval to assess FD and growth in a prospective study. Six patients in the cohort showed significant growth within 1 year. Measurements were performed in 10 planes orthogonal to the centerline of the thoracic aorta. In 1 out of 10 planes growth was significantly associated with FD. Based on these small numbers, FD was dichotomized whereby diameter growth was associated with FD of *>*5%, whereas FD of *<*5% was associated with patients that exhibited no growth.^32^

### Wall Biomarkers

All included articles concerning wall biomarkers are listed in Table 2. Only 1 of these articles included an animal model and all studies focused on AAAs.

#### Wall stiffness

Aneurysmal wall stiffness is considered to be indicative of wall strength and can be measured using MR elastography (MRE). MRE is a noninvasive MRI-based technique that uses shear wave propagation and enables the assessment of aortic stiffness in animals and humans.^9,33^ Dong et al^9^ included 31 pigs in a study involving in vivo aortic MRE and ex vivo uniaxial tensile and burst testing. To obtain the peak stress, a uniaxial tensile test is performed whereby the sample is subjected to tension until failure. During burst testing, the aorta samples were pressurized within the lumen by injecting saline into the specimen. Burst pressure was defined as the pressure at which rupture occurred. Both peak stress and burst pressure were inversely correlated with MRE-derived aortic stiffness. Based on MRE, the wall stiffness of the AAA was significantly higher compared with normal aorta (prior to creating the AAA).^9^ This result was replicated in a patient study involving aortic MRE in 24 patients with AAA and 12 healthy volunteers. Wall stiffness was significantly higher in AAA patients compared with remote nondilated aortas and healthy volunteers and was independent of the AAA diameter.^33^

#### Microvasculature in the wall

The microvasculature within the vessel wall may contribute to AAA progression by supplying inflammatory cells and matrix metalloproteinases (MMPs) which promote inflammation and extracellular matrix breakdown.^54–56^ Perfusion of the vessel wall can be assessed using dynamic contrast-enhanced (DCE) MRI. With DCE-MRI, the uptake of gadolinium-based contrast agent is visualized based on T_1_ changes in the tissue by acquiring a series of images during contrast administration. A pharmacokinetic model is used to convert the measured MR signal intensity into the contrast agent concentration and to calculate several DCE-MRI-derived parameters, such as K^trans^. K^trans^ is the volume transfer constant of gadolinium-based contrast agent from the blood vessel into the extracellular extravascular space and reflects the microvascular flow, permeability and surface area of the microvasculature.^34^ According to a study involving 28 AAA patients, DCE-MRI of the aortic vessel wall was feasible and reproducible.^34^ The same study reported a moderate correlation between K^trans^ and the maximal AAA diameter. Zhou et al^35^ included 27 male AAA patients under surveillance with DCE-MRI. They calculated the recent AAA growth rate based on 2 years of follow-up imaging with computed tomography or MRI prior to inclusion in the study. Besides K^trans^, also area under the curve (AUC) and initial enhancement slope of the signal were included as DCE-MRI-derived parameters.^35^ Growth rate was associated with slope and AUC at 4 minutes, even after controlling for maximum AAA diameter in a linear mixed effect model.^35^ Whether DCE-MRI can predict future growth was not assessed in this study.

#### Late gadolinium enhancement

In AAAs, inflammation is indicative of angiogenesis and can be identified using fluorodeoxyglucose (FDG)-positron emission tomography (PET) in combination with late gadolinium enhancement (LGE) MRI. FDG is a marker for enhanced glucose metabolism. Lesions with both LGE and FDG may be more prone to disease progression and rupture because of a higher inflammatory index. Kuzniar et al^36^ used integrated PET-MRI and included AAA patients with a diameter of at least 4.5 cm and a relatively fast expansion rate (*>*2 mm/year). Although little overlap between LGE and FDG hotspots was observed, aneurysm growth was correlated with the presence of both hotspots.^36^ Based on these results, it is not yet understood what type of cellular activity is represented by each marker and what their predictive value is.

### Molecular Biomarkers

All included studies involving molecular biomarkers are listed in Table 3. Studies on superparamagnetic particles of iron oxide ((U)SPIO) have been conducted in patients and animals with an AAA. The remaining molecular biomarkers were studied only in animal AAA models.

#### Ultrasmall superparamagnetic particles of iron oxide

Vascular inflammation is induced by the gradual infiltration of inflammatory cells, such as lymphocytes and macrophages, into the outer part of the aorta and often into the adjacent tissues.^57^ It has been suggested that inflammation plays an important role in the pathophysiology of AAA, owing to the presence of extensive inflammatory infiltration in end-stage AAAs.^58^ In addition, aneurysm-related symptoms are more frequent (65–90%) in inflammatory AAAs compared with ordinary degenerative AAAs.^2^

Magnetic resonance imaging contrast agents containing SPIO detect cellular inflammation in tissues by targeting macrophages. Ultrasmall superparamagnetic iron oxide (USPIO), with particle sizes ranging between 10 and 30 nm, can persist in the bloodstream, where they are taken up by tissue-resident macrophages.^59–62^ In a study including AAA patients scheduled for surgery, SPIO uptake was assessed in vivo in the luminal and deeper layers of thrombi. Postoperative ex vivo MR imaging was combined with thrombus analyses based on microscopy and zymography to confirm the in vivo findings.^39^ In vivo SPIO uptake was mainly localized at the luminal thrombus interface and correlated with the abundance of leukocytes.^39^ Therefore, SPIO can be used to visualize cellular inflammation. In a pilot study, USPIO uptake was assessed in 29 AAA patients by detecting a change in the T_2_* value on T_2_*-weighted images acquired before and 24 to 36 hours after USPIO administration. The MRI results were validated by histological examinations and showed that USPIO uptake was associated with macrophage infiltration, which seemed to differentiate patients with rapid and slow AAA expansion.^41^ This hypothesis was tested in a prospective multicenter cohort study including 342 AAA patients and a clinical follow-up of ≥2 years with aneurysm rupture or repair as endpoint.^40^ The investigators concluded that USPIO-enhanced MRI showed potential to predict the rate of aneurysm expansion and clinical outcomes, including rupture and death, but was not independent of known clinical risk factors such as baseline AAA diameter and smoking habit.^40^ To improve the predictive value of USPIO-enhanced MRI for AAA rupture risk, Adams et al^16^ combined USPIO and collagen-targeting probes in a murine AAA model. Their study consisted of an outcome-based longitudinal component and a week-by-week assessment of AAA development. The combination of both molecular probes was the most accurate predictor of AAA rupture. The implementation of this dual-probe is currently hindered by the collagen probe not being approved for clinical use.^16^

#### Contrast agents targeting proteolytic enzymes

##### P947 contrast agent to target MMPs

Matrix metalloproteinases in particular MMP2 and MMP9,^63,64^ are important enzymes causally linked to AAA initiation and progression.^65^ A new MRI contrast agent, P947, which targets MMP, was tested in rats with an induced AAA and compared with other contrast agents. P947 showed the highest signal enhancement in the MMP-enriched inflamed aneurysmal walls. These results were validated based on in situ zymography of histologic sections. Based on this study, P947 can be utilized to detect high-risk AAAs in rats.^37^

##### ADAMTS4-specific magnetic resonance probe

Kaufmann et al^42^ introduced a new MR probe that targets ADAMTS4 (a disintegrin and metalloproteinase with thrombospondin motifs 4) which can break down ECM and is involved in aortic aneurysm formation and rupture.^66^ This MR probe was validated in vivo in a murine model involving cross-sectional and longitudinal parts, as well as ex vivo in excised human aortic tissue. In the cross-sectional part, mice underwent MRI with an ADAMTS4-specific probe after 2 weeks and 4 weeks of angiotensin II infusion. Significant increases in the MR signal of 88 and 166% were found after 2 and 4 weeks, respectively. Next to ADAMTS4-MRI also native MRI was utilized in the longitudinal parts of the study to assess the aortic diameter. Based on the longitudinal studies it was concluded that further aortic diameter expansion could be detected by the MR probe at an early stage. In addition, based on ADAMTS4-specific MR signal enhancement, animals that underwent angiotensin II infusion for 4 days could be divided into 3 categories: development of an AAA likely to rupture, AAA development and no AAA development. Magnetic resonance signal enhancement in vivo was consistent with the ex vivo signal enhancement in 3 samples of human ruptured aortic aneurysms.^42^

#### Biomarkers targeting elastin and collagen

In the extracellular matrix, elastin and collagen are the main components responsible for maintaining structural integrity of the vessel wall.^57,67^ A decrease in elastin concentration occurs throughout the process of aneurysm growth.^67,68^ In the absence of medial elastin, adventitial collagen is responsible for the aortic wall resistance. Therefore, collagen degradation might be the ultimate cause of rupture.^69,70^

##### Collagen-specific contrast agent

Adams et al^16^ studied both a collagen 1-specific gadolinium-based probe and an iron oxide-based probe (USPIO) in a murine model as described previously. Based on T_2_-weighted imaging, the collagen-specific probe was deemed feasible for distinguishing collagen-rich aneurysms from collagen-poor aneurysms that are more prone to rupture. Similar results were obtained in another animal study using CNA-35 micelles as collagen-specific contrast agent and T_1_-weighted imaging before and 32 hours after contrast injection.^21^

##### Tropoelastin-specific contrast agent

Tropoelastin turnover may be a new biomarker of dysfunctional matrix remodeling specifically present in aortic aneurysms and can be identified using a gadolinium-based tropoelastin-specific MR contrast agent (Gd-TESMA) in an animal model. Lavin et al^38^ used a murine aortic dilatation model in a study consisting of a cross-sectional and longitudinal (4 weeks) component. They performed in vivo imaging of the murine model with Gd-TESMA with LGE MRI and T_1_ mapping, and a subsequent ex vivo validation with excised human aneurysmal tissue using MRI and histological analyses. Gd-TESMA enhancement correlated with the initiation and development of aortic dilatation. In excised human aneurysmal tissue, tropoelastin accumulation was identified using Gd-TESMA enhanced MRI, which advocates for clinical applicability.^38^

## Discussion

This scoping review provides an overview of the available quantitative MRI techniques that facilitate the assessment of aortic aneurysm progression and rupture risk by visualizing potential quantitative biomarkers. A large portion of the included studies involved hemodynamic biomarkers assessed using 4D-flow MRI. Wall biomarkers were assessed using DCE-MRI and MRE. Both these techniques are already used in other fields of medical research, which would ease clinical implementation. In particular, DCE-MRI-derived biomarkers showed potential to assess AAA growth rate. In addition, a large variety of molecular biomarkers have been studied. The ADAMTS4-specific probe could predict AAA development and rupture in a murine model. Nevertheless, as for the study concerning ADAMTS4, more than half of the molecular studies have been conducted in animal models and have not yet been tested in humans.

Based on the articles included in this review, 1 can conclude that a considerable amount of research has been conducted on different quantitative MRI techniques that visualize a variety of biomarkers targeting a variety of aneurysm initiation or exacerbation mechanisms. Nonetheless, none of the biomarkers listed in this scoping review are presented as a quantitative measure with a threshold and, therefore, are not yet ready for clinical implementation. The majority of the studies focused on accurately measuring the biomarker and relating it to the aneurysm diameter in a cross-sectional manner. Only 2 studies involving patients had a prospective design and examined the endpoints aneurysm growth, rupture or repair.^32,40^

4D-flow MRI seems a promising technique for patients with aneurysms because many potentially clinically relevant hemodynamic parameters can be derived from the resulting data. This technique has developed tremendously over the last few years and has been used in cardiac MR.^71^ However, the lack of a standardized grading scale complicates the overall interpretation of nonlaminar flow distributions visualized using 4D-flow MRI. WSS is a promising 4D flow-derived biomarker that has been investigated in multiple studies. It was inversely correlated with significant ascending aorta growth in patients in a study with 1-year follow-up.^32^ However, OSI was less promising because of inconsistent conclusions.^8,27,29^ In addition, the involvement of intraluminal thrombus, which forms a barrier between the flow lumen and endothelium, on hemodynamic parameters such as WSS is not yet clear and warrants further investigation.^72,73^

Wall biomarkers received little attention in comparison with the other 2 biomarker types. Nevertheless, 2 studies involving DCE-MRI in patients with an AAA showed promising results, whereby both AUC and slope of the signal curve were significantly correlated with aneurysm growth independent of AAA diameter. This independence means that the biomarkers could be of added value in the clinic and could strengthen clinical decision-making when used alongside aneurysm diameter. Nevertheless, only past growth was considered and whether DCE-MRI-derived parameters also correlate with future growth remains to be determined. Again the role of intraluminal thrombi should be noted because in both DCE-MRI studies, only patients with concentric intraluminal thrombi were included. The effect of thrombus on perfusion in the wall is unclear,^34^ and the effect of selection bias originating from including only patients with concentric intraluminal thrombi is unknown.

Molecular biomarkers target the visualization of specific molecular mechanisms. The main hurdle with these biomarkers, which are predominantly studied in animals, is the link between the visualized molecular mechanism and actual rupture risk in patients. This link was missing in most molecular MRI studies included in this review. In 1 study that investigated USPIO in AAA patients, a follow-up period of ≥ 2 years and endpoints rupture or repair were included. However, USPIO-enhanced MRI could not provide an independent predictor of aneurysm progression.^40^ The ADAMTS4-specific MR probe was tested in a murine model with longitudinal setup and endpoints AAA expansion and rupture. This MR probe has the potential to serve as an early marker of AAA rupture and could distinguish animals that would suffer AAA rupture from animals that would develop an AAA and those that would not.^42^ ADAMTS4-probe-related signal enhancement seems consistent in human aortic tissue; however, translation to the clinic still advocates for a study including patients. Owing to the favorable molecular composition and size of the probe, its high solubility and degradational stability, it has great potential for clinical implementation.

Other reviews regarding imaging techniques for assessing AAAs have been previously reported. Measures of local vascular characteristics of the aneurysm wall to predict AAA progression and rupture independently of AAA diameter were described in a systematic review.^74^ The authors emphasize the potential role of wall metabolism as a predictive biomarker and highlight the lack of prospective studies in the field of predictive AAA biomarkers. Another systematic review focused on molecular imaging of AAAs with PET^10^ and concluded that the available evidence is too inconsistent to determine a cut-off of ^18^F-FDG uptake to predict AAA rupture.

At this moment 3 clinical trials are ongoing involving quantitative MRI to assess AAA progression and rupture. The first study (NCT02387255) uses MRE in 3 groups: surveillance patients diagnosed with AAA (N=50), patients scheduled for surgical AAA repair (N=50), and healthy volunteers (N=60). Surveillance patients will undergo MRE every 6 months for 3 years or until: the end of the study, repair, or death due to AAA rupture or other causes. In the other groups, participants will undergo MRE once. This clinical trial has great promise to provide new information regarding the relationship between wall stiffness and AAA progression and rupture. Another ongoing clinical trial (NCT04811222) investigates the use of a radiotracer probe in PET-MRI called gallium-dotatate, specific for activated macrophages inducing inflammation. The study design is cross-sectional, with retrospective diameter surveillance data, including 55 AAA patients who will undergo 1 PET-MRI scan. The last clinical trial (NCT05976711) just started in our own institute and includes surveillance AAA patients (N=20) who will undergo 2 MRI scans involving 4D-flow MRI and DCE-MRI with a half year interval.

### Recommendations for Future Research

Future studies should first focus on acquiring longitudinal imaging data from cohorts that involve patients with aneurysm growth, repair, and/or rupture. In this way, the potential causal relationship between the biomarkers discussed in this review and aneurysm endpoints can be determined and their reproducibility assessed. A good example is the study by Perera et al,^75^ who studied several hemodynamic and morphological biomarkers in both ruptured and unruptured intracranial aneurysms and found significant differences between the groups. Similarly, independent predictors of aortic aneurysm rupture can also be assessed. These longitudinal studies would require large cohorts because only a minority of aortic aneurysm rupture. The relatively low incidence of aneurysm rupture results in difficulty in gaining statistical power, as seen in the study with the largest cohort of all studies included in this review.^40^ Setting up an imaging biobank for all AAA and TAA patients under surveillance may be a solution to this problem. To facilitate clinical implementation, the established quantitative biomarkers should be categorized using thresholds. Ultimately, we could work toward a multifactorial quantitative rupture risk score that takes both morphological parameters, such as maximum diameter and dynamic quantitative biomarkers derived from MRI, into account. An example of a multifactorial score for intracranial aneurysms without dynamic biomarkers is the PHASES (Population, Hypertension, Age, Size of aneurysm, Earlier subarachnoid hemorrhage from another aneurysm, Site of aneurysm) score.^76^ Such a score with integrated quantitative biomarkers could facilitate a more complete overview of the risks and patient-specific aortic aneurysm management in the future.

### Limitations

The studies included in this review used different subjects to test their potential biomarkers, such as animal models, healthy volunteers and patients with different types of aortic aneurysms, and underlying diseases. As a result, the state of development of the presented biomarkers varies, which complicates their comparison. In addition, both thoracic and AAAs were studied in the included articles. Compared with AAAs, TAA have different pathogenic, biomechanical, and histological features.^77,78^ Therefore, results from thoracic studies are not directly interchangeable with AAAs. Nevertheless, involving thoracic research in this review is vital because it provides additional valuable information in the search for quantitative imaging biomarkers. Moreover, we consider TAA research to be informative for AAA research, and vice versa, and therefore including both is vital to create a complete picture. Finally, no critical appraisal of the included articles was performed because this was complicated by the inclusion of studies with several different study designs and subjects and it is not necessitated according to the PRISMA extension for scoping reviews.^23^

## Conclusion

This scoping review provides an overview of quantitative MRI techniques and the range of biomarkers derived from these techniques for the assessment of aortic aneurysm progression and rupture risk. Found biomarkers were subdivided into hemodynamic, wall, and molecular biomarkers. Longitudinal studies with aneurysm growth, rupture, or repair as endpoints are scarce, although they are required to validate potential biomarkers. Although further studies are required, quantitative MRI could play an important role in personalized risk assessment of aortic aneurysm rupture, facilitating the development of a multifactorial score, including diameter, to assess aortic aneurysm progression.

## Appendix

### Search in PubMed

(((magnetic resonance imaging[MeSH Terms]) OR (magnetic resonance[Title]) OR (MR[Title]) OR (MRI[Title])) AND ((Aortic Aneurysm[MeSH Terms]) OR (thoracic aortic aneurysm*[Title/Abstract]) OR (abdominal aortic aneurysm*[Title/Abstract]) OR (aorta[Title/Abstract]) OR (aortic aneurysm*[Title/Abstract]) OR (AAA[Title/Abstract]) OR (TAA[Title/Abstract]) OR (aneurysm*[Title/Abstract])) AND ((functional* Title/Abstract) OR (quanti*[Title/Abstract]) OR (fmri[Title/Abstract]) OR (qmri[Title/Abstract]) OR (quantitative magnetic resonance imaging[Title/Abstract]) OR (functional magnetic resonance imaging[Title/Abstract]) OR (4D flow[Title/Abstract]) OR (dynamic[Title/Abstract]) OR (molecular MRI[Title/Abstract])) AND ((aorta[Title/Abstract]) OR (aneurysm*[Title/Abstract]))) NOT ((Case Reports[Publication Type]) OR (Editorial[Publication Type])) AND (2010:2023[pdat])
